# Oxidative Stress and Bronchial Asthma in Children—Causes or Consequences?

**DOI:** 10.3389/fped.2017.00162

**Published:** 2017-07-24

**Authors:** Milos Jesenak, Maria Zelieskova, Eva Babusikova

**Affiliations:** ^1^Jessenius Faculty of Medicine, Department of Pediatrics, Comenius University in Bratislava, University Hospital, Martin, Slovakia; ^2^Jessenius Faculty of Medicine, Department of Medical Biochemistry, Comenius University in Bratislava, Martin, Slovakia

**Keywords:** bronchial asthma, oxidative stress, oxidative damage of biomolecules, chronic inflammation, childhood

## Abstract

Bronchial asthma is one of the most common chronic inflammatory diseases of the airways. In the pathogenesis of this disease, the interplay among the genes, intrinsic, and extrinsic factors are crucial. Various combinations of the involved factors determine and modify the final clinical phenotype/endotype of asthma. Oxidative stress results from an imbalance between the production of reactive oxygen species and reactive nitrogen species and the capacity of antioxidant defense mechanisms. It was shown that oxidative damage of biomolecules is strongly involved in the asthmatic inflammation. It is evident that asthma is accompanied by oxidative stress in the airways and in the systemic circulation. The oxidative stress is more pronounced during the acute exacerbation or allergen challenge. On the other hand, the genetic variations in the genes for anti-oxidative and pro-oxidative enzymes are variably associated with various asthmatic subtypes. Whether oxidative stress is the consequence of, or the cause for, chronic changes in asthmatic airways is still being discussed. Contribution of oxidative stress to asthma pathology remains at least partially controversial, since antioxidant interventions have proven rather unsuccessful. According to current knowledge, the relationship between oxidative stress and asthmatic inflammation is bidirectional, and genetic predisposition could modify the balance between these two positions—oxidative stress as a cause for or consequence of asthmatic inflammation.

## Introduction

Bronchial asthma (BA) represents the most common chronic respiratory disease in children, and its prevalence is constantly increasing especially in the developing countries (1). The disease is characterized by chronic ongoing airways inflammation accompanied by structural (so-called remodeling changes—epithelial fragility, goblet cell hyperplasia, enlargement of submucosal mucus glands, hypertrophy and hyperplasia of airway smooth muscles, airway wall thickening) and functional changes (specific and non-specific hyperresponsiveness, quantitative and qualitative changes in mucus production) in the airway wall. BA develops as a consequence of the interaction among genes (disease determining and modifying genes), intrinsic (e.g., hormonal changes, immune dysregulation) and extrinsic (e.g., allergens, pollutants, infections, physical and environmental factors) factors. The intrinsic and extrinsic factors are able to modify the gene expression directly or indirectly through epigenetic changes (2, 3).

Recently, many attempts were made to classify and stratify the diseases into different phenotypes or endotypes, which are characterized by specific patterns and aspects with respect to the development of inflammation. Phenotype means a cluster of characteristics that define asthma and its subsets. Till date, several asthmatic phenotypes have been determined and characterized based on the triggers (e.g., allergen-induced asthma, non-allergic asthma, infections-exacerbated asthma, aspirin-exacerbated respiratory diseases, exercise-induced asthma) or clinical presentation (e.g., transient wheezing, non-atopic wheezing in toddlers, exacerbation-prone asthma) (4). Another classifying approach is represented by the endotypes of asthma, which attempts to characterize asthma subset according to the pathophysiological mechanisms involved in the development and persistence of asthmatic inflammation in the airways (5). There were several differences between children and adults regarding BA pathophysiology and clinical manifestations (6). The interplay between immune and non-immune cells leads to inflammation, which is characterized by various levels of clinical expression and symptoms. During the pre-exacerbation, the acceleration of the inflammatory cascades results in the asthma exacerbation of different severities (7). The persistent and intermittently exacerbated inflammation is accompanied by overproduction of reactive oxygen species (ROS) and reactive nitrogen species (RNS), which can also contribute to the promotion and persistence of the airway inflammation (8).

## Oxidative Stress and BA Development

### Oxidative Stress in Health and Disease

Oxidative stress was shown to be an important component of the aging processes (9) and many other pathological conditions and processes. Oxidative stress results from an imbalance between the production of pro-oxidants (e.g., ROS or RNS) and antioxidant defense mechanisms in the body. The endogenous sources of ROS are cell organelles (mitochondria, peroxisomes, endoplasmic reticulum), various enzymes and enzymatic complexes (e.g., cytochrome P_450_, NADPH oxidases, nitric oxide synthase, xanthine oxidase), immune and non-immune cells (especially phagocytes, activated eosinophils and neutrophils, monocytes and macrophages, airway epithelial and smooth muscle cells, endothelium), and others (e.g., heme proteins, reactions of metal ions). On the other hand, there are also many exogenous sources of ROS, such as cigarette smoke, ultraviolet light, ionizing radiation, pollutants, ozone, organic solvents, metals, and some medicaments (e.g., chemotherapeutic agents). Both endogenous and exogenous sources of ROS can play an important role in the pathogenesis and worsening of various inflammatory conditions, especially through continual accumulation of the oxidative changes in biomolecules (8, 10–13).

### Mechanisms of Oxidative Stress in BA

Many authors showed that BA is significantly associated with increased oxidative stress expressed by the increased markers of oxidative damage. Based on the development and persistence of oxidative stress, several crucial aspects and mechanisms can be identified (11):

- Overproduction of ROS and RNS in chronic inflammation (13, 14).
- Deficiency of intrinsic (e.g., glutathione) or extrinsic (e.g., vitamins and natural antioxidants in diet) antioxidant substances (15–17).
- Decreased activity or dysfunction of antioxidant enzymes (18, 19).
- Over-activity of pro-oxidative enzymes (20–22).

Production of highly reactive oxygen species leads to the progressive damage of various biomolecules (nucleic acids, lipids, proteins, saccharides) with the functional and structural consequences. Under physiological condition, antioxidant defense mechanisms are able to eliminate and repair these changes. However, unregulated overproduction of ROS, during inflammation of various origins, leads to accumulation of the changes without sufficient repair. Oxidative damage of the biomolecules influences the signaling pathways, enzymatic functions, gene expression, and many other essential biological processes. Structural changes are contributing to the chronic remodeling of the tissues. Another possible contribution to the oxidative damage of biomolecules in addition to ROS is RNS. These are formed from the reaction of nitric oxide with oxygen or ROS. RNS could modify the thiol groups (–SH groups; formation of nitrosothiol) or change the tyrosyl residues to nitrotyrosine. This process usually leads to the inactivation or dysfunction of the modified proteins (10, 23).

### Oxidative Stress and BA—Molecular Consequences

**Figure 1:**
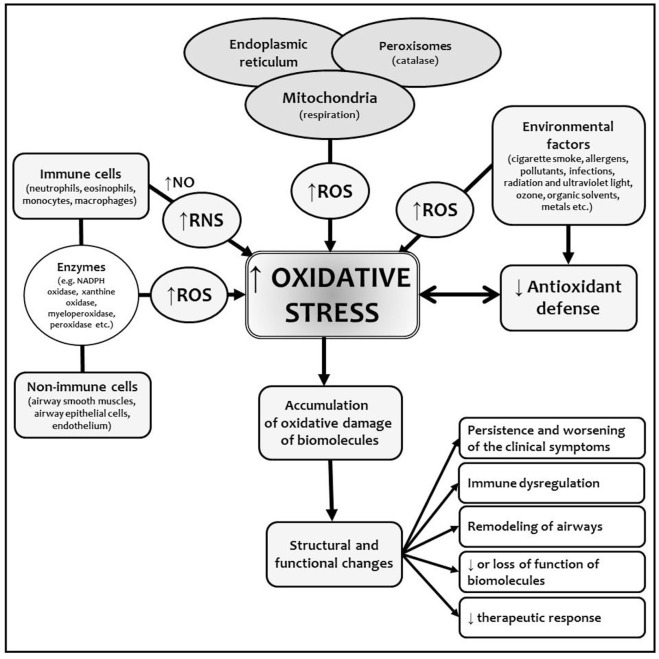
Origin of ROS and RNS and oxidative stress in bronchial asthma (BA). Chronic inflammation in BA is characterized by the overproduction of ROS/RNS as a consequence of increased activation of immune and non-immune cells in cellular inflammatory infiltrate. Various exogenous factors promote and amplify the inflammation and also increased the formation of reactive species *via* many mechanisms (e.g., induction of mitochondrial dysfunction, DNA repair mechanisms damage). Chronic inflammation decreases the capacity of endogenous antioxidant defense mechanisms, which are not able to compensate overproduction of ROS/RNS. This leads to the accumulation of the changes in biomolecules with structural and functional consequences. Abbreviations: NO, nitric oxide; RNS, reactive nitrogen species; ROS, reactive oxygen species.

Oxidative stress represents a substantial component of BA development and persistence. Chronic inflammation leads to the overproduction of ROS and RNS. During the allergen challenge or other situations (i.e., infection, pollutants, physical endurance), the pronouncement of inflammatory cascades is accompanied by the accelerated production of ROS/RNS. This could contribute to the structural changes in the airways, support the remodeling processes, decrease the sensitivity to the anti-inflammatory and anti-asthmatic treatment, and worsen the clinical course of the diseases. Uncontrolled asthma with the sinusoidal acceleration of ROS/RNS production pronounces the pathophysiological aspects of the inflammation and dysfunction of the biomolecules and is clinically associated with remitting–relapsing course of the disease (Figure 1) (8, 12, 23).

**Figure 2:**
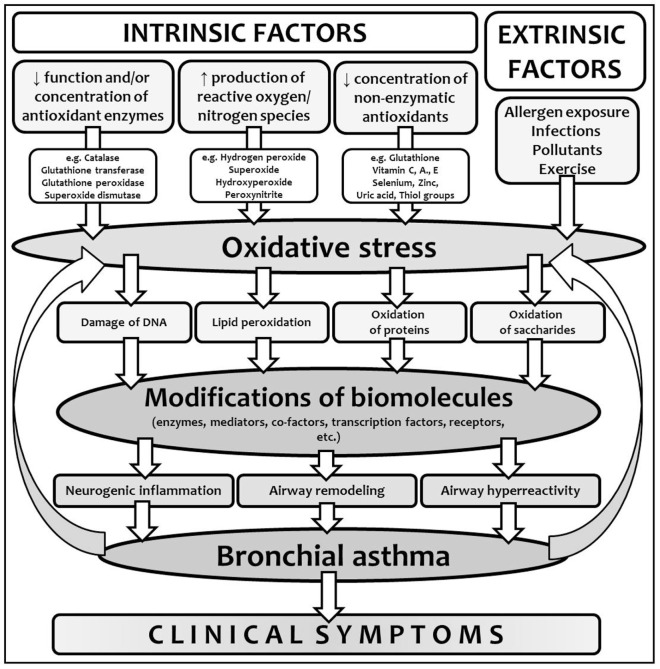
Oxidative stress in the context of bronchial asthma development. Interplay between intrinsic and extrinsic factors leads to the production of reactive oxygen species (ROS) and in certain conditions, due to the imbalance between the production of ROS and antioxidant defense, oxidative stress and modification of biomolecules develops. Due to the modifications of various biomolecules with different functions, particular components of chronic inflammation lead to asthma development and clinical symptoms onset. Moreover, non-controlled or partially controlled and regulated inflammation is another important source of ROS, which closes the vicious circle.

Many studies showed that the cells formatting airway inflammation in BA are an important source of ROS in these patients (23). Oxidative damage of the biomolecules could have both functional and structural consequences. It plays an important role in the development, persistence, and consequences of BA of all phenotypes or endotypes, since inflammation is crucial in pathogenesis of all the asthmatic forms and subtypes. ROS and RNS, insufficient function of antioxidant defense mechanisms and oxidation of the biomolecules have many consequences, such as enhanced release of arachidonic acid from cell membranes and formation of inflammatory markers, hyperreactivity and contraction of airway smooth muscle, increased vascular permeability with airway edema, increased bronchial hyperresponsiveness and mucus secretion, increased synthesis of pro-inflammatory cytokines and chemoattractants, induced release of tachykinins and neurokinins with augmentation of neurogenic inflammation, and impaired response to bronchodilators (Figure 2) (8, 11, 16).

Exposure to environmental allergens has been shown to stimulate overproduction of ROS/RNS, resulting in the damage of DNA (nuclear and mitochondrial), proteins, and lipids with decrease or even loss of their physiological functions. Moreover, accumulation of oxidative damage causes the structural changes in the airways tissues (16). It was also shown that environmental allergens, such as house dust mite-allergens, possess direct cytotoxicity and lead to the increased oxidative damage, and DNA double-strand breaks in asthmatic lungs (24). Moreover, oxidative damage of DNA and histones could lead to epigenetic changes, such as the diminished responsivity to anti-asthmatic drugs, e.g., corticosteroids (21). On the other hand, the ROS production still presents an important part of adaptive and protective mechanisms.

### Markers of Oxidative Stress in BA

Due to low concentrations of ROS, their extreme reactivity, and very short lifetime, direct measurement of ROS is very complicated and, therefore, various indirect markers of oxidative damage, such as markers of lipid peroxidation (e.g., malondialdehyde, 8-isoprostane, thiobarbituric acid-reactive substances (TBARS), acrolein, hexanal, heptanal, nonanal, 4-hydroxyhexanal, 4-hydroxynonenal), protein oxidative damage (e.g., decreased content of free thiol groups, nitrotyrosine, nitrosothiols), DNA damage (e.g., 8-hydroxy-2-deoxyguanosine), or general total antioxidant capacity of plasma could be used (25). Oxidative stress and its manifestations have been shown to be present both in the airways (bronchoalveolar lavage fluid, airway tissue, epithelial lining fluid, exhaled breath condensate, sputum, saliva) and in the systemic circulation (25–30). Airway oxidative stress in BA could be attributed to various sources of ROS, such as exposure to environmental pro-oxidants, airway infiltration of inflammatory immune cells, metabolic dysregulation, and reduced capacity of antioxidant mechanisms (12). Increased production of ROS during airway inflammation is more pronounced after the allergen challenge (31). It was suggested that the assessment of oxidative stress by-products can be used for asthma severity monitoring (16).

It was shown that oxidative stress markers in BA are associated with worse clinical control, worse disease severity, or reduced lung function (32). The amount of ROS is directly correlated with the degree of bronchial hyperreactivity (14). The most impressive changes can be observed during acute exacerbation of asthma, in asthma with concomitant allergic rhinitis or in poorly controlled asthma. Changes in the concentration of the antioxidants and the markers of oxidative damage can be determined in the peripheral blood, serum, plasma, bronchoalveolar lavage fluid, lung, and bronchial tissues biopsies and even in the exhaled breath and its condensate (25–30, 33–37). Clusterin represents a sensitive cellular biosensor of oxidative stress with antioxidant properties and is associated with the clearance of cellular debritus and apoptosis. Its serum concentration is elevated in patients with severe asthma and is inversely correlated with lung functions (38).

Interestingly, umbilical cord blood-derived basophils from the neonates born to mother with atopic asthma showed increased markers of oxidative stress, decreased activity of glutathione peroxidase, and increased production of interleukin 4. This could contribute to the development of allergic hyperreactivity in children at risk of asthma (39).

## Antioxidant Strategies for Asthma Treatment

Based on the observation from the previous studies that BA is associated with decreased antioxidant protection due to changes in the functions of antioxidant enzymes of decreased concentrations of non-enzymatic antioxidants, the use of different antioxidants could be an interesting mode of supportive and complementary anti-asthmatic therapy (10, 28).

Epidemiological data suggest that antioxidants have a significant effect on the incidence and severity of BA. It was shown that BA and airflow limitation are associated with deficiency of various antioxidants, such as carotenoids, retinol, coenzyme Q10, and vitamin C, D, and E (17, 40–45). In many studies, total antioxidant status of the serum in asthmatics was lower when compared with healthy controls (28, 46). Conversely, the antioxidant deficiencies were not confirmed in other studies (47).

In several studies, the supplementation with a nutraceutical of antioxidants and anti-inflammatory compounds (e.g., curcumin, zinc, selenium, vitamin D) was associated with reduction of airway inflammation, as documented by a decrease in fractional exhaled nitric oxide (FENO) (48) or prolonged time to exacerbation (49). Supplementation of coenzyme Q10 reduced the dosage of oral corticosteroids in steroid-dependent asthmatics (50). Conversely, consumption of broccoli sprouts did not improve eosinophilic inflammation, inflammatory or oxidative stress markers, or other clinical features of asthma among atopic asthmatics despite a marked increase in the serum levels of sulforaphane, which is a potent inducer of antioxidant enzymes (51). Recently, porous antioxidant polymer microparticles were developed as a potential therapeutic system for BA. They possess antioxidant activity and could be used as a carrier for anti-asthmatic drugs, e.g., corticosteroids (52). However, other studies did not confirm the general benefits from antioxidant supplementation in asthma management (53). There are no clinical trial data to support the use of antioxidants to prevent asthma or allergy development (54). Other than corticosteroids, which possess antioxidant and anti-inflammatory properties, other anti-asthmatic drugs, e.g., montelukast, did show conflicting results regarding their capacity to improve total antioxidant status and decrease oxidative damage (55, 56).

Tobacco smoke exposure was confirmed to be an important risk factor for bronchial hyperreactivity development. It increases the oxidative stress with its further consequences. Moreover, it was shown that toxic chemicals in tobacco smoke are able to induce epigenetic changes with increased expression of various pro-inflammatory genes (57). Therefore, prenatal and postnatal exclusion of tobacco smoke exposure could be used as an important preventive approach against allergic sensitization and inflammation development with the attenuation of oxidative stress (58). Prenatal supplementation of vitamin C and E improved pulmonary functions of newborns of smoking mothers (59), but in a large placebo-controlled study, this effect was not confirmed (60).

Oxidative stress is a dynamic process with very tiny border between protective and harmful effects and with problematic predictability of the threshold after which disease ensues and, therefore, this could explain a general inconsistence in the clinical trials with antioxidant supplementation in asthma management. Although the supplementation of various antioxidants appears to be a promising adjuvant therapy for asthma, various studies did not confirm the significant benefits over standard therapy. The potential of antioxidant therapy could be improved by taking into consideration individual characteristics of each particular asthmatic (e.g., the presence of various polymorphisms in the genes for antioxidant enzymes) and environmental risk factors, instead of treating oxidative stress in the airways broadly (12, 41).

## Summary

In our research, we studied and analyzed the possible role of oxidative damage in the development of BA in children from several aspects. We studied the markers of oxidative stress in exhaled breath [exhaled carbon monoxide (eCO), nitric oxide] and in the peripheral blood (concentration of free thiol groups as a marker of protein oxidation and concentration of TBARS as a marker of lipid peroxidation) and also analyzed the selected single nucleotide polymorphisms in the genes for two important antioxidant enzymes, such as polymorphisms of two important antioxidant enzymes, theta isoform of glutathione transferase (gene *GST-T1*) and catalase (gene *CAT*).

Examination of exhaled breath seems to be very practical tool for investigation and clinical management of BA both in children and in adults. Till date, several molecules were detected in exhaled breath of asthmatics, but the most commonly used marker of airway inflammation is the FENO. In the group of our asthmatic children, we clearly showed positive correlation between the levels of FENO and the concentration of total IgE in serum and peripheral blood eosinophils. Atopic asthmatics, children with concomitant allergic rhinitis and asthmatics yielded higher levels of FENO during acute exacerbation when compared with non-atopic asthmatics, children without allergic diseases of upper airways and during the stable, clinically controlled disease, respectively (34). Although FENO is preferentially considered to be the marker of allergic and eosinophilic airway inflammation, on the other hand, it can also reflect the increasing oxidative stress (61). Moreover, nitric oxide could also react with oxygen or ROS to form RNS, such as peroxynitrite, which contribute to the increased oxidative damage under pathological conditions. Therefore, FENO could also be used as an indirect marker of oxidative stress. The eCO is another non-invasive marker detectable in exhaled breath under different pathological conditions of respiratory tract. Exhaled CO is produced by heme oxygenase 1, whose activity can be increased by different factors, such as ROS. Therefore, eCO can serve not only as an indirect inflammatory marker but also as a marker of oxidative stress (15). We showed that eCO is higher in asthmatic children when compared with healthy subjects. Acute exacerbation of BA is accompanied by a significant increase in eCO compared to clinically controlled disease. The level of eCO is higher in atopic asthmatics when compared with non-atopic asthmatics and asthma associated with allergic rhinitis. Moreover, we studied the correlation between selected markers of oxidative damage of biomolecules and eCO levels. We found a significant negative correlation between the concentration of free thiol groups and eCO in atopic asthmatics and during acute exacerbation of asthma. Since we were not able to find such correlation in non-atopic asthmatics, in controlled asthma and in healthy subjects, it can be assumed that eCO could be used as an indirect marker of oxidative stress in different respiratory tract diseases, such as BA (35).

As discussed above, oxidative stress and oxidative damage of the biomolecules represents an important part of chronic inflammation in the airways. We confirmed decreased concentration of free thiol groups (marker of protein oxidation) in asthmatic children compared to healthy subjects. Atopics showed significantly decreased concentration of –SH groups compared to non-atopic asthmatics. We also evaluated a marker of lipid peroxidation—concentration of TBARS. Asthmatics yielded higher concentration of TBARS than healthy controls, especially of atopic phenotype and during acute asthmatic exacerbation (18, 62). Besides the markers of oxidative damage of proteins and lipids, we also focused on the analysis of the selected polymorphisms of two important antioxidant enzymes—theta isoform of glutathione transferase (gene *GST-T1*) and catalase. These two enzymes represent essential mechanisms in the protection against oxidative damage, because they utilize a wide range of products of oxidative damage as substrates. Children with asthma had higher prevalence of the *GST-T1* null genotype compared with healthy controls and these genotypes increased the risk for asthma development by 3.17 folds. Interestingly, atopic asthmatics had a lower prevalence of *GST-T1* gene null genotype than non-atopics (62). Regarding the selected polymorphism -262 C/T in catalase gene (*CAT*), the TT genotype was more frequent in asthmatics than in healthy children (OR = 5.63). This genotype correlated positively with the concentration of –SH groups and negatively with the content of TBARS (18).

## Conclusion

Oxidative stress represents an important part in the pathogenesis of asthma, but on the other hand, it is only a part in the complex mosaic of BA development. Contribution of oxidative stress to asthma pathology remains at least partially controversial, since antioxidant interventions have proven rather unsuccessful. According to current knowledge, it is not possible to definitely resolve whether oxidative stress is the reason or a consequence of chronic inflammation in asthmatic airways. Since the therapeutic use of antioxidants was not generally proven in clinical studies for asthma, the appropriate selection of asthmatic patients with the potential to benefit from antioxidant therapy needs further investigation. Future research should be focused on the detection of the individual asthmatics, in which application of a particular antioxidant strategy could modify the clinical course and support the standard medication. Moreover, the development of effective antioxidant therapy (new antioxidants or modification in the existing anti-asthmatic molecules with increased antioxidant properties) with complex biological effects could improve the clinical management of asthmatic and allergic patients. Another issue in the context of preventive allergology would be the development of preventive antioxidant strategies that would help in the global burden of allergic diseases.

## Author Contributions

All the authors equally contributed to the concept of the work, performed literature search, written the text, revised it critically, and approved the final version of the manuscript to be published.

## Conflict of Interest Statement

The authors declare that the research was conducted in the absence of any commercial or financial relationships that could be construed as a potential conflict of interest.
